# Low Affinity DnaA-ATP Recognition Sites in *E. coli oriC* Make Non-equivalent and Growth Rate-Dependent Contributions to the Regulated Timing of Chromosome Replication

**DOI:** 10.3389/fmicb.2018.01673

**Published:** 2018-07-26

**Authors:** Prassanna Rao, Tania A. Rozgaja, Abdulaziz Alqahtani, Julia E. Grimwade, Alan C. Leonard

**Affiliations:** ^1^Department of Biological Sciences, Florida Institute of Technology, Melbourne, FL, United States; ^2^AREVA Med, Plano, TX, United States

**Keywords:** *oriC*, DnaA, DNA replication, replication origin, orisomes, pre-replicative complexes, DNA binding proteins, cell cycle

## Abstract

Although the mechanisms that precisely time initiation of chromosome replication in bacteria remain unclear, most clock models are based on accumulation of the active initiator protein, DnaA-ATP. During each cell division cycle, sufficient DnaA-ATP must become available to interact with a distinct set of low affinity recognition sites in the unique chromosomal replication origin, *oriC*, and assemble the pre-replicative complex (orisome) that unwinds origin DNA and helps load the replicative helicase. The low affinity *oriC*-DnaA-ATP interactions are required for the orisome’s mechanical functions, and may also play a role in timing of new rounds of DNA synthesis. To further examine this possibility, we constructed chromosomal *oriC*s with equal preference for DnaA-ADP or DnaA-ATP at one or more low affinity recognition sites, thereby lowering the DnaA-ATP requirement for orisome assembly, and measured the effect of the mutations on cell cycle timing of DNA synthesis. Under slow growth conditions, mutation of any one of the six low affinity DnaA-ATP sites in chromosomal *oriC* resulted in initiation earlier in the cell cycle, but the shift was not equivalent for every recognition site. Mutation of τ2 caused a greater change in initiation age, suggesting its occupation by DnaA-ATP is a temporal bottleneck during orisome assembly. In contrast, during rapid growth, all origins with a single mutated site displayed wild-type initiation timing. Based on these observations, we propose that *E. coli* uses two different, DnaA-ATP-dependent initiation timing mechanisms; a slow growth timer that is directly coupled to individual site occupation, and a fast growth timer comprising DnaA-ATP and additional factors that regulate DnaA access to *oriC*. Analysis of origins with paired mutated sites suggests that Fis is an important component of the fast growth timing mechanism.

## Introduction

All cells must initiate new rounds of chromosomal DNA synthesis with temporal precision and only once per cell cycle to avoid genome instability. Bacteria accomplish this by using a triggering mechanism that requires the AAA+ (ATPases Associated with various cellular Activities) initiator protein, DnaA. In cells, DnaA is bound to either ATP or ADP, and DnaA-ATP is considered to be the active form of the initiator based on *in vitro* unwinding and replication assays (Sekimizu et al., 1987). Multiple DnaA molecules interact with distinct high and low affinity recognition sites in the unique chromosomal replication origin (*oriC*) to assemble a pre-replicative complex (orisome) that unwinds origin DNA and then assists with loading the replicative helicase onto the single strands (reviewed in Bell and Kaguni, 2013; Leonard and Grimwade, 2015; Chodavarapu and Kaguni, 2016).

**FIGURE 1:**
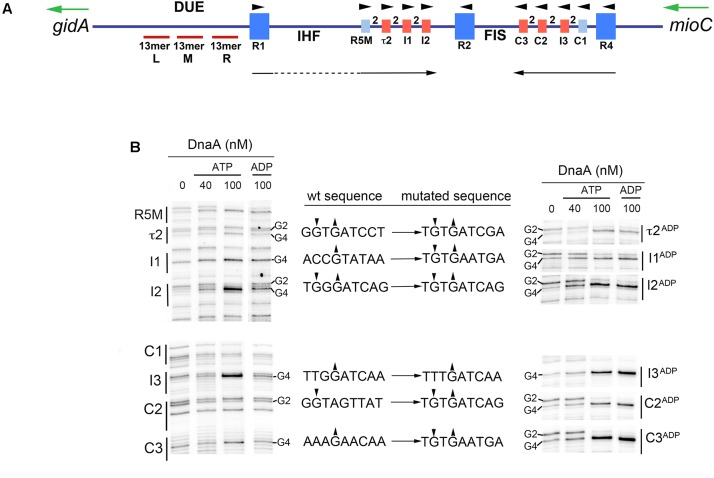
Six low affinity DnaA-ATP sites in *E. coli oriC* can be converted to sites that bind DnaA-ADP. **(A)** Map of *E. coli oriC*, showing low affinity DnaA recognition sites that preferentially bind DnaA-ATP (red boxes), and high and low affinity sites that bind both nucleotide forms (dark and light blue boxes, respectively). The number 2 between boxes indicates the spacing between sites. The positions of the binding sites for Fis and IHF are shown, as well as the left (L), middle (M), and right (R) 13mer repeats in the DNA Unwinding Element (DUE) are indicated. Arrowheads indicate the orientation of the binding sites. Black arrows indicate the direction of progressive DnaA binding. Green arrows indicate the direction of transcription of the genes flanking *oriC* (*gidA* and *mioC*). Figure is from Leonard and Grimwade (2015). **(B)** Comparison of DnaA-ATP and DnaA-ADP binding to wild type *oriC* and *oriC* containing the indicated mutations. DMS footprints, showing binding of purified his-tagged DnaA-ATP (40 and 100 nM) and DnaA-ADP (100 nM) are shown. All the binding sites are marked, with positions of the guanosine residues in the second and fourth positions (if present) indicated. Only the footprint of the mutated site on each mutated plasmid is shown in the right panel. Small arrowheads indicate enhancement (up arrow) or suppressions (down arrow) of DMS modification. Intervening lanes on the gels have been removed in the figure, and regions between R1 and R5M, and C3 and R2 were also removed.

In *E. coli oriC*, there are 11 DnaA binding sites within the 190 base pairs that are adjacent to the DNA Unwinding Element (DUE) (**Figure 1A**; Bramhill and Kornberg, 1988; Kowalski and Eddy, 1989; Rozgaja et al., 2011). Three of these sites (R1, R2, and R4), with consensus sequence, 5′-TTA/TTNCACA-3′, bind DnaA with high affinity. The remaining eight sites deviate at two or more positions and bind DnaA with low affinity (**Figure 1A**), reviewed in Leonard and Grimwade (2011), Leonard and Méchali (2013), and Wolański et al. (2014). These weak binding sites lie in the gap regions between R1 and R2, and R2 and R4, with each gap containing a cluster of four sites separated by 2 bp (**Figure 1A**). All low affinity DnaA recognition sites in *oriC* are incapable of binding DnaA directly (Schaper and Messer, 1995); instead, their occupation requires the DnaA occupying high affinity sites (usually R1 and R4) to recruit and donate DnaA to the nearest low affinity site (usually R5M and C1), followed by cooperative DnaA binding between the arrayed low affinity sites, so that DnaA occupation progresses toward the center of *oriC* (Simmons et al., 2003; Miller et al., 2009; Rozgaja et al., 2011).

Two DNA bending proteins, IHF and Fis, modulate the low affinity DnaA interactions in *oriC*. IHF-catalyzed bending of the DNA between R1 and R5M places these two sites into proximity, facilitating cooperative binding in *trans*, and thus beginning the progressive DnaA occupation of the left half of *oriC* (Grimwade et al., 2000; Rozgaja et al., 2011). However, IHF binding to its recognition site is prohibited as long as Fis occupies its recognition site between R2 and C3, and higher levels of DnaA are required for orisome assembly if the *oriC* template is occupied by Fis (Ryan et al., 2004). Fis is bound to *oriC* throughout most of the cell cycle, but is displaced by the progressive DnaA binding extending from R4 (Ryan et al., 2004); loss of Fis relieves the repression of IHF binding and allows rapid DnaA occupation of any unbound low affinity sites. This dynamic interplay among DnaA, Fis, and IHF ensures synchronous initiations from the multiple *oriC* copies that exist during rapid growth (Weigel et al., 2001; Ryan et al., 2004). However, because Fis is a growth rate-regulated protein (Nilsson et al., 1992; Mallik et al., 2006) it is not likely to be an orisome component in slowly growing cells, and without Fis-mediated inhibition, IHF is likely to be bound thought the cell cycle.

Despite our ever-increasing understanding of orisome assembly and regulation (Leonard and Grimwade, 2011; Skarstad and Katayama, 2013; Katayama et al., 2017; Schenk et al., 2017), it remains unclear how replication initiation is precisely coupled to the bacterial cell division cycle. Most models for initiation timing (Mahaffy and Zyskind, 1989; Hansen et al., 1991b; Browning et al., 2004; Grant et al., 2011; Zhang and Shi, 2012) are based on accumulation of DnaA-ATP, whose levels fluctuate during the cell cycle, peaking near the time of initiation (Kurokawa et al., 1999). There is extensive experimental evidence in support of these models (Atlung et al., 1987; Pierucci et al., 1987; Løbner-Olesen et al., 1989; Skarstad et al., 1989; Atlung and Hansen, 1993), but some controversy remains (Xu and Bremer, 1988; Flåtten et al., 2015). In addition, most models have yet to take into account the manner in which DnaA-ATP interacts with *oriC*. The three high affinity sites bind DnaA-ATP and DnaA-ADP equivalently (McGarry et al., 2004), and remain occupied throughout the cell cycle (Samitt et al., 1989; Nievera et al., 2006). For this reason, occupation of these sites is unlikely to be the rate-limiting step in triggering chromosome replication. Instead, it is more probable that initiation timing is determined by the filling of the low affinity DnaA recognition sites because occupation of these sites is transient and takes place immediately before initiation (Nievera et al., 2006; Rozgaja et al., 2011). Further, although sites C1 and R5M have equal preference for DnaA-ATP and DnaA–ADP, all remaining low affinity sites (τ2, I1, I2, and C3, C2, and I3) exhibit a 4-fold binding preference for DnaA-ATP (McGarry et al., 2004; Grimwade et al., 2018), suggesting that occupation of the sites with DnaA-ATP could couple initiation to levels of available DnaA-ATP.

Site preference or non-preference for DnaA-ATP is determined by nucleotide sequence, and previous work demonstrates that *oriC* remains functional when some or all low affinity sites are altered by mutagenesis to allow both DnaA-ATP and -ADP forms to bind equivalently without changing overall site affinity (McGarry et al., 2004; Grimwade et al., 2018). DnaA is not normally degraded after bound ATP is hydrolyzed, and DnaA-ADP is prevalent in cells, fluctuating between 30 and 70% of total DnaA (Kurokawa et al., 1999). Since mixed complexes of DnaA-ATP and DnaA-ADP can activate *oriC* (Yung et al., 1990; Grimwade et al., 2007, 2018), if DnaA-ADP can access *oriC* at mutated sites, this binding should reduce the requirement for DnaA-ATP in orisome formation, and shift the timing of initiation earlier in the cell cycle. Previous studies support this idea. For example, although cloned wild-type *oriC*, initiates synchronously with the wild-type chromosomal copy (Helmstetter and Leonard, 1987; Koppes, 1987; Løbner-Olesen, 1999), *oriC* plasmids carrying loss of preference mutations at I2 and I3 rapidly integrate and replace chromosomal *oriC*, consistent with the cloned origin initiating earlier and out-competing the chromosomal origin (Grimwade et al., 2007). Further, changing all the DnaA-ATP sites in chromosomal *oriC* into sites that bind both DnaA-ATP and DnaA-ADP causes over-initiation and loss of initiation synchrony (Grimwade et al., 2018), similar to the phenotype of cells in which there is a 3–4-fold excess of DnaA (Atlung and Hansen, 1993). However, it remains unclear whether all DnaA-ATP sites play equivalent timing roles (i.e., each site requiring a fixed amount of time to become occupied as newly synthesized DnaA-ATP becomes available) or if additional factors might contribute to how DnaA accesses the origin sites.

To better understand the relationship between low affinity site occupation and the initiation clock, we measured the timing of DNA synthesis in cells whose only chromosomal *oriC* copy carried mutations resulting in loss of DnaA-ATP preference at individual or pairs of low affinity recognition sites. We observed that during slow growth, the onset of chromosomal DNA replication was shifted to earlier cell cycle times for all single site mutations, but the shift was greater in cells carrying mutations at the τ2 site, revealing a possible bottleneck step during orisome assembly. Initiation timing in cells where pairs of sites were mutated was consistent with this idea. However, altering DnaA-ATP preference at single sites did not affect timing when assays were performed under rapid growth conditions. These observations are consistent with the existence of two distinct, growth-rate dependent timing mechanisms in *E. coli*, and we propose that the occupation of *oriC* low affinity DnaA-ATP recognition sites directly regulates initiation timing only during slow growth. In rapidly growing cells, although initiation is not triggered until all the low affinity sites are occupied, other factors modulate binding of DnaA-ATP to these sites. Examination of double site mutations during fast-growth suggests that one of these factors is the growth rate-regulated protein Fis, making this architectural protein an important component of the initiation timing mechanism.

## Materials and Methods

### Chemicals, Proteins, Enzymes, and Oligonucleotides

Reagent grade chemicals were purchased from IBI, ThermoFisher, or Sigma. Media components were from IBI or Difco. All enzymes were from New England Biolabs. Oligonucleotides were purchased from ThermoFisher. His-tagged DnaA was isolated as described (Li and Crooke, 1999), including a urea wash step to remove bound nucleotide and lipids.

### Construction of *oriC*s With Loss of DnaA-ATP Preference

The positions of mutated DnaA recognition sites are shown in **Figure 1A**. Specific changes in nucleotide sequence are listed in **Figure 1B**. Site-directed mutagenesis was performed as described in McGarry et al. (2004). The starting template for all *oriC* mutations was pOC170 (3,852 bp) (Langer et al., 1996), which carries both the pBR322 replication origin and *oriC*. Overlapping 30–35 bp primers carrying the base alterations were flanked by 10 bp of DNA homologous to the template. To make mutations in multiple DnaA binding sites in *oriC*, a single site was mutated per reaction, and the resulting plasmid used as template to make additional mutations. Supercoiled plasmid was isolated using a plasmid preparation kit (IBI). Mutations in *oriC* were confirmed by sequence analysis (sequencing primer was 5′-CTCAACTTTGTCGGCTTGAG) and plasmids were introduced into DH5α by transformation selecting for ampicillin resistance.

### Recombineering

PCR fragments carrying the mutant *oriC* were amplified from plasmids using primers near *gidA* (5′-CACGGCCACCGCTGTAATTAT) and *mioC* (5′-ATCCCATACTTTTCCACAGG). The fragments were introduced by electroporation into ACL402 (*asnB32, relA1, spoT1, thi-1, fuc-1, lysA, ilv-192, zia*::pKN500, Δ*dnaA, mad-1*, λcI857Δ(*cro-bioA*), *nad*::Tn10, Δ*oriC::cat-sacB*) following induction of RED gene expression, as described in Kaur et al. (2014). ACL402 was constructed by inserting the cat-*sacB* cassette from pK03 (Link et al., 1997) into ACL401 (Kaur et al., 2014). Transformed cells were grown in LB overnight at 32°C without selection, and then diluted and plated onto LB agar plates containing 5% sucrose and incubated at 32°C overnight. Replacement of the Δ*oriC::cat sac* with the mutated *oriC* was verified in sucrose resistant cells by testing for loss of chloramphenicol resistance, and nucleotide sequence analysis. The *oriC* region from verified mutant cells was transduced using P1 phage into an JEG22, an MG1655 derivative which is *asnA*::cat and *asnB*::Tn10 and transductants were selected for growth in the absence of asparagine on minimal salts agar (Helmstetter and Krajewski, 1982), supplemented with 0.1% glucose as described in Kaur et al. (2014).

### Dimethyl Sulfate Footprinting

DMS modification of DNA (0.75 μg) *in vitro* was performed as described (Ryan et al., 2004). DnaA was pre-incubated in reaction buffer with 5 mM ATP or ADP for 5 min before addition to reactions at the concentrations indicated in **Figure 1B**. DMS-treated samples were extended with radiolabeled primer as described (Ryan et al., 2004). Primer sequences were 5′-GTATACAGATCGTGCGATC, for revealing R1-I2, and 5′-GGATCATTAACTGTGAATG, for revealing R4-R2. Extension products were resolved on 6% polyacrylamide sequencing gels, and dried gels were scanned on a Bio-Rad Molecular Imager FX PhosphorImager. Representative scans are shown in **Figure 1B**.

### Western Blots

MG1655 was grown for at least five generations in exponential growth, in minimal media containing 0.1% uracil supplemented with 0.1% glucose plus 0.1% casamino acids; 0.1% glucose; 0.2% glycerol; or 0.4% succinate. Samples were removed from the cultures, pelleted, and flash frozen in liquid nitrogen three times to lyse the cells. Protein concentrations in each sample were quantified using the MicroBCA assay (Pierce) and then equal amounts of protein (5 mg) were resolved on 12% PAGE/SDS gels. The proteins were transferred to nitrocellulose, and DnaA and Fis on the blots were revealed using anti-DnaA and anti-Fis antibodies with detection by chemiluminescence (Bio-Rad ImunoStar kit). Assays were done in triplicate, with representative scans shown in Supplementary Figure S1.

### Flow Cytometry

Wild-type or mutant *oriC* strains (MG1655 derivatives) were grown in minimal salts media (Helmstetter and Krajewski, 1982; Løbner-Olesen et al., 1989) supplemented with 20 μg/ml uracil and either 0.4% succinate or 0.1% glucose, 0.1% casamino acids, and 0.1% uracil, and liquid cultures were grown to exponential phase at 37°C with vigorous shaking. Cells were then treated with 300 μg/ml rifampicin and 15 μg/ml cephalexin (Skarstad et al., 1986; Løbner-Olesen et al., 1989), and incubation was continued at 37°C for at least 4 h. After drug treatment, 1 ml of cells was fixed with 9 ml of 70% ethanol and stored at 4°C. Prior to flow cytometric analysis, 1 ml of the fixed cell suspension was pelleted, washed with 50 mM Tris-Cl, pH 7.5, 150 mM NaCl (TBS), and resuspended in 1 ml of TBS containing 0.5 μl/ml Vybrant DyeCycle Green (ThermoFisher) (final concentration of dye is 2.5 μM). Stained cells (3,000–5,000 cells/ml) were analyzed using an Accuri C6 personal flow cytometer, and data from 10,000 cells were collected. Forward scatter was used for cell mass measurement. CFlow software was used to calculate the percentage of cells in each chromosomal DNA peak.

## Results

### Conversion of a DnaA-ATP Site Into One That Binds DnaA-ADP Alters Initiation Timing in Slowly Growing *E. coli* Cells

DnaA-ADP is produced in bacterial cells by hydrolysis of DnaA-ATP, which, in *E. coli*, is stimulated by the Hda protein associated with replication forks, as well as by the *datA* locus, reviewed in Katayama et al. (2017). Since *E. coli* cells do not degrade DnaA-ADP, this form is generally available for *oriC* interactions at any recognition sites capable of binding it. Therefore, mutation of a low affinity DnaA-ATP recognition site to allow interaction with DnaA-ADP should decrease the amount of DnaA-ATP required for orisome formation, and, if the mutation is carried in chromosomal *oriC*, it should shift initiation timing to earlier in the cell cycle. To examine this possibility, we first expanded our collection of site mutants (McGarry et al., 2004; Grimwade et al., 2007, 2018) by altering each of the six individual DnaA-ATP recognition sites (see **Figures 1A,B**), as well as pairs of sites in *oriC* specifically chosen for their location (two sites on the right, two on the left, and one in each half). Since we wished to retain the low affinity attribute while allowing DnaA-ADP binding, the I3, C2, C3, I2 I1, and τ2 sites were each individually mutated to sequences identical or similar to R5M. The sequence of the mutated sites is shown in **Figure 1B**. To verify that the mutations relieved the DnaA-ATP preference, but did not alter overall site affinity, binding of DnaA-ATP or DnaA-ADP to each site was evaluated using dimethyl sulfate (DMS) footprinting (**Figure 1B**). DnaA was pre-incubated with nucleotide, and then bound to supercoiled *oriC* plasmid prior to DMS treatment and primer extension. Sites that are occupied by DnaA are revealed by distinctive changes to the DMS modification pattern resolved on sequencing gels. Specifically, the G4 of the 9 mer consensus 5′-TGTGGATAA (or variations of this sequence) becomes hypersensitive, and the G2 less sensitive to DMS (**Figure 1B**), causing darker and lighter bands, respectively. The changes in band intensity increase as all of the plasmid molecules in the treatment population become occupied. It should be noted that not all binding sites have guanosines at both positions. For evaluation of site affinity, two concentrations of DnaA-ATP were used: 40 nM, which is insufficient for full occupation of all low affinity sites, and 100 nM, which previous studies have shown to be a saturating concentration, resulting in complete DnaA-ATP binding to all *oriC* copies in the reaction mix (McGarry et al., 2004; Grimwade et al., 2007). In wild type *oriC* (**Figure 1B**, left panel), the pattern of DnaA-ATP binding is consistent with previous studies showing R1, R2, and R4 as higher affinity sites fully occupied at 40 nM DnaA, and the remaining sites being fully occupied only at the higher concentration. The DnaA-ATP binding pattern is similar for origins containing the mutated sites, indicating that the mutations do not alter any sites’ affinity for DnaA-ATP. However, when DnaA-ADP is used in the reaction, only the mutant origin is fully occupied by 100 nM DnaA-ADP (**Figure 1B**); in contrast, binding of 100 nM DnaA-ADP binding to I3, C2, C3, I2 I1, and τ2 in wild type *oriC* gives a DMS modification pattern similar to that seen with 40 nM DnaA-ATP (McGarry et al., 2004).

We next evaluated the effect of single site mutations on initiation timing. A previous study reported that mutations may affect chromosomal *oriC* differently than *oriC*’s harbored on a plasmid (Weigel et al., 2001). Therefore, recombineering (homologous recombination-mediated genetic engineering) was used to delete wild-type *oriC* on the chromosome (Court et al., 2002), and replace it at the native location with one of the mutant *oriC*s carrying an altered low affinity site. After verifying successful origin transplantation by sequence analysis, P1 phage was then used to transfer the mutated origin from the recombineering strain into MG1655 for analysis of cell cycle timing. In the initial experiments, *E. coli* containing wild type or mutant *oriC* were grown in minimal media supplemented with succinate as a carbon source (generation time 101–103 min). This growth rate allows us to examine the simplest initiation scenario, in which only one *oriC* copy per cell must be activated, since the generation time of *E. coli* growing in this media should be greater than the time needed to complete chromosome replication and divide (∼60 min), so exponentially growing cells will contain only one copy of *oriC* prior to the time of initiation (Cooper and Helmstetter, 1968). Further, since previous studies by our lab revealed that Fis, when bound to *oriC*, reduces binding of DnaA to low affinity sites, and because Fis is a growth-rate regulated protein, orisome assembly in cells growing in succinate media should take place without Fis inhibition. To confirm this, equal amounts of total protein from cells with wild type *oriC*, growing exponentially at four different growth rates were separated on denaturing polyacrylamide gels, transferred to nitrocellulose, and the blots were incubated with both anti-DnaA and anti-Fis antibodies (Supplementary Figure S1). The blots confirm that Fis levels decrease as a function of growth rate (Flåtten and Skarstad, 2013), and are nearly undetectable in cells using succinate as a carbon source, while DnaA concentrations remain constant among the growth rates tested, as has been reported previously (Hansen et al., 1991a).

**FIGURE 2:**
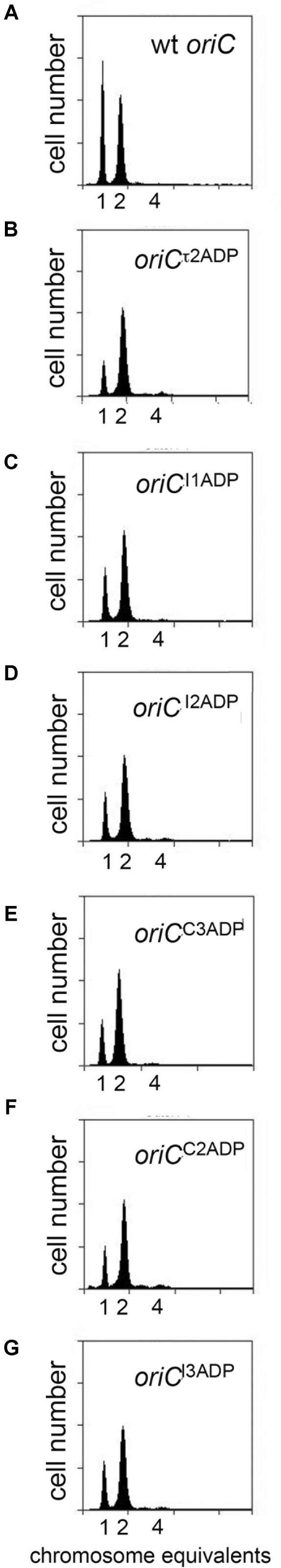
Mutation of individual DnaA-ATP sites into sequences that equivalently bind DnaA-ADP causes a shift in initiation timing in slowly growing *E. coli* cells. **(A–G)** DNA histograms of cells with wild type *oriC* or with *oriC*s carrying the indicated single site mutations, growing in minimal media using succinate as the carbon source were treated with cephalexin and rifampicin, and analyzed by flow cytometry. The number of chromosome equivalents, corresponding to the number of origins at the time of drug treatment, is shown.

To evaluate initiation timing, cells carrying the mutated *oriC*s were grown to mid-exponential phase in minimal media supplemented with succinate. Then, cells were treated with rifampicin to inhibit new rounds of chromosome replication, and cephalexin, which prevents cell division, followed by incubation to allow completion of ongoing rounds of replication. After staining cellular DNA with a fluorescent dye, the number of chromosomes in the cell, which reflects the number of *oriC* copies present at the time of drug addition, was detected by flow cytometry. The origin number doubles at the time of initiation, and at all growth rates, only 2^n^ origins should be in these cells. Accordingly, cultures of MG1655 cells contained 1 or 2 origins (**Figure 2A**), and initiated ∼36 min after cell division (**Table 1**). Although previous studies report that cells growing slowly with acetate as the carbon source display rifampicin resistant initiations (Flåtten et al., 2009), we do not see evidence of this in cells growing in succinate media, since the percentage of cells that have not yet started chromosome replication (1 chromosome peak) does not decrease after addition of rifampicin (Supplementary Figure S2). Interestingly, converting a single DnaA-ATP site into one that binds both of DnaA’s nucleotide forms changed initiation timing in all the mutant cells, resulting in an increased number of origins per cell, and an earlier initiation time in the cell cycle (**Figures 2B–G** and **Table 1**). The conversion of the τ2 site to τ2^ADP^ caused a greater change in timing (**Table 1**), suggesting that filling this site might represent a rate-limiting step in slow growth orisome formation.

**Table 1:** Doubling times, number of origins, and cell mass of strains carrying mutated sites growing in minimal media supplemented with succinate.

Site mutated	Doubling time (min)	Number of origins/cell	Relative cell mass^a^	Origins/cell mass	Ai^b^
None	102	1.57	1.0	1.57	0.35 (36 min)
τ2^ADP^	101	2.00	1.0	2.00	0.13 (13 min)
I1^ADP^	102	1.81	1.0	1.81	0.18 (18 min)
I2^ADP^	103	1.82	1.0	1.82	0.17 (18 min)
C2^ADP^	102	1.81	1.0	1.81	0.18 (18 min)
C3^ADP^	102	1.84	1.0	1.84	0.18 (18 min)
I3^ADP^	103	1.80	1.0	1.80	0.18 (18 min)
τ2/I2^ADP^	102	2.70	1.0	2.70	0.05 (5 min)
τ2/I3^ADP^	101	2.05	1.0	2.05	0.11 (11 min)
I3/C3^ADP^	102	1.83	1.0	1.83	0.18 (18 min)
I2/I3^ADP^	102	2.00	1.0	2.00	0.13 (13 min)


*^a^Relative cell mass was measured by flow cytometry, using forward scattered light of mutant strain relative to the wild type strain.*

**FIGURE 3:**
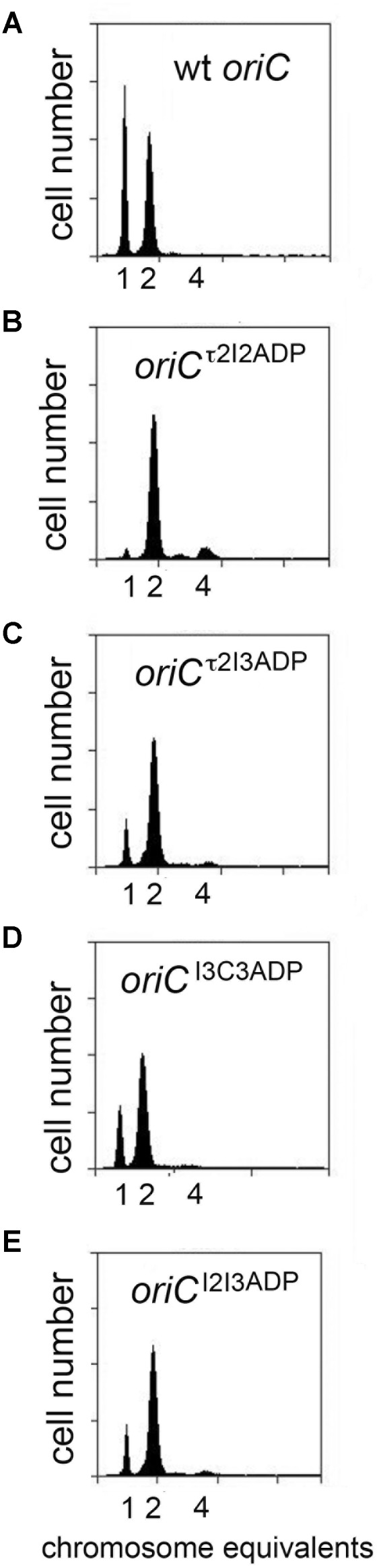
Initiation timing of slowly growing *E. coli* cells carrying two DnaA-ATP site mutations in *oriC*. **(A–E)** DNA histograms of cells with wild type *oriC* or with *oriC*s carrying the indicated two site mutations, growing in minimal media supplemented with succinate, were treated with cephalexin and rifampicin, and analyzed by flow cytometry. The number of chromosome equivalents, corresponding to the number of origins at the time of drug treatment, is shown.

*^b^Ai (age at initiation), as a fraction of the cell cycle generation time, was calculated using the formula Ai = -ln(1-F/2)/ln2 where F is the fraction of cells in a population that have not initiated chromosome replication, measured from flow cytometric histograms of cells treated with rifampicin and cephalexin (**Figures 2, 3**). The calculated Ai was multiplied by the generation time to give age of initiation in minutes.*

The change in initiation time caused by converting one DnaA-ATP site to the non-discriminatory form raised the possibility that changing more than one site might have an additive effect. To evaluate this, we examined the effect of mutation pairs on initiation during slow growth. Mutation pairs chosen for study were located either in *oriC*’s right half (I3/C3), left half (τ2/I2) or each half (τ2/I3; I2/I3), because there is evidence that DnaA assembled onto the left and right halves of *oriC* play different roles in initiation (Ozaki et al., 2012), and we wished to examine if the two sub-assemblies made equivalent contributions to setting the time of initiation. Accordingly, cells carrying the double mutations were grown in minimal media supplemented with succinate, treated with rifampicin and cephalexin, and the number of chromosome equivalents, corresponding the number of origins at the time of drug treatment, was determined by flow cytometry (**Figure 3**). The effect of mutating two sites in the right sub-assembly (I3^ADP^ and C3^ADP^) did not change timing appreciably from that obtained for cells containing these mutations individually (**Figure 3D** and **Table 1**). In contrast, placing two mutations (τ2^ADP^ and I2^ADP^) in the left sub-assembly shifted the time of initiation much more than either of the single mutations (**Figure 3B** and **Table 1**). Initiation timing in cells harboring a mutated site in both left and right assemblies appeared to be determined largely by the left half mutation, with the τ2^ADP^/I3^ADP^ mutant cells initiating slightly earlier that the I2^ADP^/I3^ADP^ cells (**Figures 3C,E** and **Table 1**), consistent with the idea that τ2 plays a greater timing role than I2. These results remain consistent with models suggesting that the right and left sub-assemblies are not equivalent (Ozaki et al., 2012).

### Individual Low Affinity DnaA-ATP Sites Do Not Play an Initiation Timing Role in Rapidly Growing Cells

**FIGURE 4:**
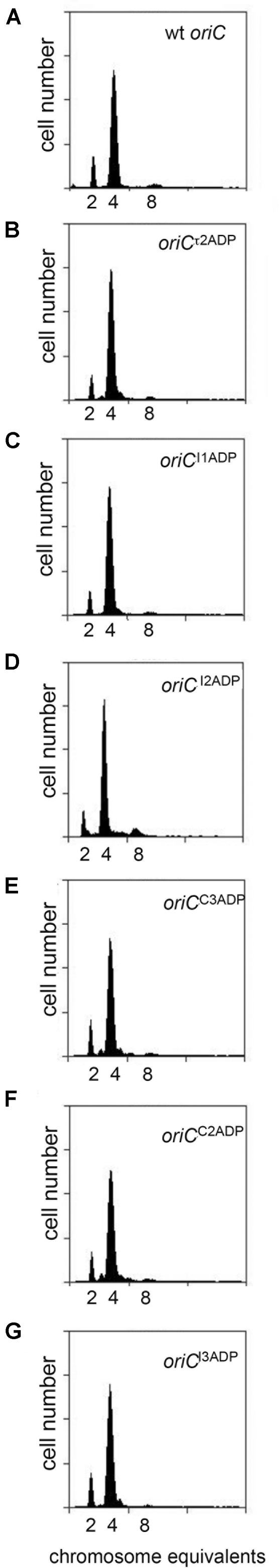
Mutation of individual DnaA-ATP sites into sequences that bind DnaA-ADP equivalently does not change initiation timing in rapidly growing *E. coli* cells. **(A–G)** DNA histograms of cells with wild type *oriC* or with *oriC*s carrying the indicated single site mutations, growing in minimal media supplemented with glucose and casamino acids were treated with cephalexin and rifampicin, and analyzed by flow cytometry. The number of chromosome equivalents, corresponding to the number of origins at the time of drug treatment, is shown.

Although the studies described above show that mutations causing loss of preference for DnaA-ATP shift initiations to earlier cell cycle times in slow growing cells, Riber et al. previously reported that the same mutations in I2 or I3 had no effect on initiation timing in rapidly growing cells (Riber et al., 2009), suggesting that the timing role of individual DnaA-ATP sites might be dependent on growth rate. To examine this possibility, we evaluated initiation timing of the six single DnaA-ATP site mutations in cells growing in minimal media supplemented with glucose and Casamino acids, with doubling times of ∼33 min. Exponentially growing cells were treated with rifampicin and cephalexin, and DNA content was measured by flow cytometry after incubation to allow completion of ongoing rounds of DNA replication. Under these growth conditions, the *E. coli* generation time is less than the time needed to complete chromosome replication, and exponentially growing cells normally contain more than one copy of *oriC*, and all origin copies initiate chromosome replication synchronously, once per cell cycle, on partially duplicated chromosomes (Cooper and Helmstetter, 1968; Skarstad et al., 1986). Accordingly, cultures of MG1655 cells contained 2, 4, or 8 origins (**Figure 4A**), and initiated ∼3 min after division. When cells containing origins carrying single site mutations were examined, we found that initiation timing was identical to that observed in cells with wild type *oriC* (**Figures 4B–G** and **Table 2**), in contrast to the shift in timing seen at the slower growth rate. These results confirm previous reports (Riber et al., 2009) and suggest that another factor may contribute to initiation timing regulation in rapidly growing cells.

**Table 2:** Doubling times, number of origins, and cell mass of strains carrying mutated sites growing in minimal media supplemented with glucose and casamino acids.

Site mutated	Doubling time (min)	Number of origins/cell	Relative cell mass^a^	Origins/cell mass	Ai^b^
None	33	4.20	1.0	4.20	0.1 (3 min)
τ2^ADP^	33	4.23	1.0	4.23	0.1 (3 min)
I1^ADP^	34	4.21	1.0	4.21	0.1 (3 min)
I2^ADP^	33	4.20	1.0	4.20	0.1 (3 min)
C2^ADP^	34	4.19	1.0	4.19	0.1 (3 min)
C3^ADP^	32	4.20	1.0	4.20	0.1 (3 min)
I3^ADP^	33	4.20	1.0	4.20	0.1 (3 min)
τ2/I2^ADP^	34	4.68	1.0	4.68	—
τ2/I3^ADP^	34	4.55	1.0	4.55	—
I3/C3^ADP^	33	3.40	1.0	3.40	—
I2/I3^ADP^	33	4.20	1.0	4.20	0.1 (3 min)


*^a^Relative cell mass was measured by flow cytometry, using forward scattered light of mutant strain relative to the wild type strain*

**FIGURE 5:**
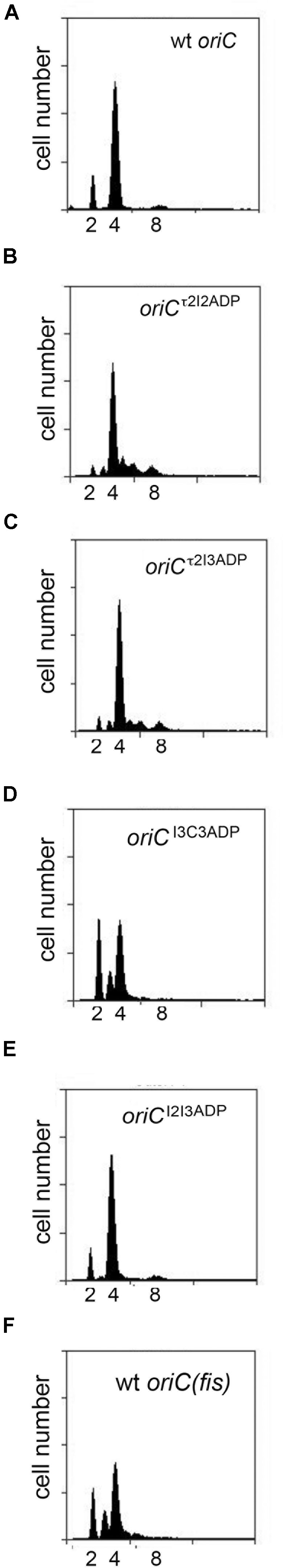
Initiation timing of rapidly growing *E. coli* cells carrying two DnaA-ATP site mutations in *oriC*. **(A–E)** DNA histograms of cells with wild type *oriC* or with *oriC*s carrying the two indicated site mutations **(F)** growing in minimal media supplemented with glucose and casamino acids were treated with cephalexin and rifampicin, and analyzed by flow cytometry. The number of chromosome equivalents, corresponding to the number origins at the time of drug treatment, is shown.

*^b^Ai (age at initiation), as a fraction of the cell cycle generation time, was calculated from cultures with synchronous initiations using the formula Ai = -ln(1-F/2)/ln2 where F is the fraction of cells in a population that have not initiated chromosome replication, measured from flow cytometric histograms of cells treated with rifampicin and cephalexin (**Figures 4, 5**). The calculated Ai was multiplied by the generation time to give age of initiation in minutes.*

We also used flow cytometry to examine DNA content in cells containing two site mutations to determine if this might alter timing (**Figure 5**). As was done in the studies of slow growing cells, the double mutants were selected to allow examination of left and right sub-assemblies (e.g., τ2/I2, I3/C3, τ2/I3, and I2/I3). Interestingly, changing both τ2 and I2 in the left half of *oriC* was sufficient to perturb initiation (**Figure 5B**), but the alteration in timing was more complex than the simple shift to earlier times seen in slower growing cells. While most of the cells in the population had 4 origins, odd numbers of origins were also detected, indicating that initiations in these cells were no longer synchronous (**Figure 5B**). The number of origins per cell was also slightly increased (**Table 2**). A similar pattern of asynchronous initiations was seen when τ2 was mutated in the left sub-assembly and I3 was mutated in the right sub-assembly (**Figure 5C** and **Table 2**). However, origins carrying both the I2^ADP^ (left) and I3^ADP^ (right) mutations showed essentially normal initiation timing (**Figure 5E**), as was previously reported (Riber et al., 2009). These results support idea that τ2 plays a greater role in initiation timing than the other sites.

Unlike the slight over-initiation seen in cells with origins carrying τ2^ADP^/I2^ADP^ and τ2^ADP^/I3^ADP^ mutations, cells with *oriC*^I3ADP/C3ADP^ contained fewer origin copies per cell, with a larger percentage of cells containing 2 or 3 origins, and a lower number of cells containing 8 origins (**Figure 5D** and **Table 2**). These results reveal a clear difference in the response to changes in the left and right halves of *oriC*. One possible cause of this difference is the role of the right DnaA sub-assembly in displacing Fis (Ryan et al., 2004). While further studies are needed to verify this idea, it is intriguing to note that the pattern of initiation in cells lacking Fis is similar to the I3^ADP^/C3^ADP^ mutant (**Figure 5F**).

## Discussion

An important question about the regulation of DNA replication during the *E. coli* cell cycle is whether low affinity DnaA recognition sites in *oriC* are components of the timing mechanism. There are several possible roles for recognition sites in a clock model based on the availability of DnaA-ATP. In one simple scenario, each low affinity recognition site would require an equivalent amount of time to become occupied. Alternatively, the timer might be based on site occupation, but sites could play a non-equivalent role, i.e., some sites would require more or less time to become occupied than others. A third possibility is that despite a requirement to fill sites with DnaA-ATP during orisome assembly, some other factor acts as the initiation timer by restricting or facilitating DnaA-ATP access to *oriC*, thus setting the DnaA-ATP level for site occupation and origin activation. Since previous DnaA over-expression studies both support and oppose DnaA’s role in initiation timing (Churchward et al., 1983; Atlung et al., 1987; Pierucci et al., 1987; Xu and Bremer, 1988; Løbner-Olesen et al., 1989; Skarstad et al., 1989; Atlung and Hansen, 1993; Flåtten et al., 2015), our approach was to examine minimally disturbed cells with altered chromosomal *oriC* low affinity DnaA recognition sites that bind to DnaA-ATP or DnaA-ADP equivalently. Any differences in DNA replication during the cell cycle compared to the wild-type version of *oriC* should implicate the altered site as a timing component.

**FIGURE 6:**
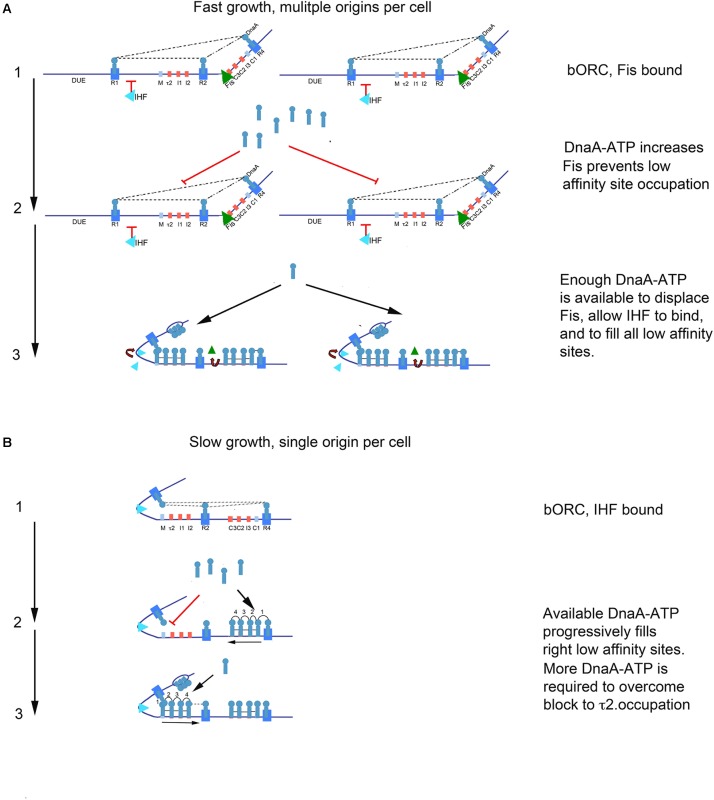
Model of growth-rate dependent regulation of DnaA-ATP site occupation during orisome assembly in *E. coli*. **(A)** In rapidly growing cells, DnaA-ATP site occupation is regulated by Fis. Stage 1 (bacterial Origin Recognition Complex; bORC): Cells contain more than one copy of *oriC*, and DnaA is bound to high affinity R1, R2, and R4 sites throughout most of the cell cycle. Fis is also bound at this stage, and inhibits IHF from occupying its site. Dotted lines indicate putative interactions between R1, R2, and R4. Low affinity sites are unoccupied. Stage 2: Bound Fis prevents accumulating DnaA-ATP from occupying low affinity sites. Stage 3: When enough DnaA-ATP accumulates and becomes available, Fis is displaced. Loss of Fis allows IHF to bind to *oriC*, and also allows all low affinity sites on all *oriC* copies in the cell to become occupied by DnaA. The sharp bend induced by IHF binding allows DnaA bound to R1 to assist loading of R5M and the left array of DnaA-ATP sites. In this configuration, *oriC* DNA is unwound in the DUE, and DnaA-ATP in the form of a compact filament binds to the ssDNA. **(B)** In slowly growing cells, DnaA-ATP site occupation is determined by the availability of DnaA-ATP, and the ability to occupy the τ2 site. Stage 1: Cells contain only one copy of *oriC*, and DnaA is bound to high affinity sites throughout most of the cell cycle. IHF is also bound at this stage. Dotted lines indicate possible interaction between DnaA bound at R1, R2, and R4. Stage 2:As DnaA-ATP become available, it progressively binds right-side low affinity sites, nucleated by DnaA bound to R4. Occupation of the low affinity sites in the left half of *oriC* is delayed by a bottleneck in filling the τ2 site. When enough DnaA-ATP has accumulated to overcome this bottleneck, *oriC* DNA is unwound in the DUE, and DnaA-ATP in the form of a compact filament binds to the ssDNA.

We obtained evidence that loss of DnaA-ATP preference at *oriC* low affinity recognition sites can shift initiation timing during the cell cycle. However, the timing role for recognition sites was more complex than expected, and we identify a dependence on both recognition site location and cellular growth rate. During slow growth in minimal medium with a succinate carbon source, we found that removing DnaA-ATP preference at any of the low affinity recognition sites caused a shift in initiation timing, supporting a site filling-dependent clock mechanism where sites fill when a molecule of DnaA-ATP becomes available to them. But, the unexpectedly larger change in initiation time caused by mutations at τ2 suggests that occupation of this site acts as a bottleneck step during orisome assembly. Although we can only speculate about the mechanism responsible, we note that filling the arrayed low affinity sites in *oriC*’s left half requires IHF-stabilized DNA bending to position the DnaA occupying R1 close enough to R5(M) and τ2 to allow cross-strand DnaA donation (Rozgaja et al., 2011; Ozaki and Katayama, 2012; Kaur et al., 2014; Shimizu et al., 2016; **Figure 6**). It is possible that some structural aspect of this orisome assembly step could impede DnaA-ATP access to τ2. It is also possible that cross-strand interactions between DnaA-ATP bound to τ2 and R1 are required to stabilize an origin configuration that promotes DnaA binding to the unwound DUE (Ozaki and Katayama, 2012; Sakiyama et al., 2017), and achieving this configuration may be more time consuming than lateral cooperative DnaA-ATP binding between arrayed sites.

Any bottleneck in orisome assembly functioning during slow growth would also be expected to function during the synchronous initiations of multiple *oriC* copies during rapid growth. However, none of the single site loss of preference mutations changed initiation timing in fast growing *E. coli*, suggesting that there could be a fast growth-specific mechanism that controls DnaA-ATP availability or its access to *oriC*. One likely component of this mechanism is the growth rate-regulated protein, Fis. We previously reported that binding of purified Fis protein to *oriC* increases the amount of DnaA-ATP required to assemble orisomes *in vitro* (Ryan et al., 2004), with displacement of Fis allowing IHF binding and immediate filling of unoccupied recognition sites by the excess DnaA (Ryan et al., 2004; Leonard and Grimwade, 2011). It is reasonable to expect an analogous situation exists *in vivo*. If the level of DnaA-ATP required to displace Fis from *oriC* over-shoots the amount needed to fill all remaining low affinity DnaA-ATP recognition sites, initiation timing would be determined by how long it takes to accumulate enough DnaA-ATP to displace Fis (see **Figure 6**). In this model, Fis serves as both a negative and positive regulator during orisome assembly, negatively regulating IHF-dependent orisome assembly steps, but positively regulating synchronous initiation by ensuring enough DnaA-ATP accumulates to fire all *oriC* copies (Ryan et al., 2004; Flåtten and Skarstad, 2013). In support of this model, we observed that mutations allowing DnaA-ADP binding at two low affinity recognition sites in the right half of *oriC* caused under-initiation as well as initiation asynchrony (**Figure 5D**). This is the expected result if the mutations resulted in Fis displacement at lower DnaA-ATP levels than would be required for synchronous initiations at all *oriC* copies. Interestingly, there is more than one Fis recognition site in the right half of *oriC* (Filutowicz et al., 1992; Hengen et al., 2003), and it is possible that the amount of accumulated DnaA needed to displace Fis might change over a range of growth rates. There may also be additional mechanisms, such as DnaA acetylation (Zhang et al., 2016; Li et al., 2017), that regulate DnaA access to *oriC* and initiation timing at very slow growth rates, where Fis is absent. Support for this idea comes from reports that *E. coli* cells using acetate as the sole carbon source have higher DnaA/*oriC* ratios than expected (Flåtten et al., 2009). It should also be noted that other DNA binding proteins can contribute to initiation timing by blocking DNA access, changing topology, or changing the availability of DnaA-ATP, reviewed in Riber et al. (2016), and it is possible that some of these could also be growth rate-dependent.

Taken together, our results suggest that *E. coli* may not use a single initiation timing mechanism at all growth rates. Instead, it appears that there are multiple timing mechanisms, each optimized for particular growth conditions, with the ability to shift between them if nutrient availability changes. Timing mechanisms might also vary among bacterial types, depending on their particular lifestyles, and this may be one reason for the large variety in the number and positioning of DnaA recognition sites seen in different bacterial replication origins (Leonard and Méchali, 2013). Although it remains to be determined whether low affinity recognition sites with DnaA-ATP preference are ubiquitous among *oriC*s (Charbon and Lobner-Olesen, 2011), variations in high affinity recognition site number and position may also result in bottlenecks and/or growth rate regulators similar to those that we suggest for *E. coli*.

Finally, it is worth noting that the change in initiation age that we observe for our mutant *oriC*s during slow growth suggests that while there must be free DnaA-ADP available to interact with recognition sites during the majority of the cell cycle, supporting previous findings for rapidly growing cells (Kurokawa et al., 1999; Grimwade et al., 2018), the availability of DnaA-ATP must be stringently restricted even near the time of initiation, such that each low affinity DnaA-ATP site fills only when a DnaA-ATP molecule becomes available. Our ability to remove the preference for DnaA-ATP from low affinity recognition sites in *oriC* and retain orisome activity also demonstrates the functional equivalence of the active and inactive (DnaA-ADP) form of the initiator for most of the steps required for orisome assembly, as long as DnaA-ADP can access the appropriate recognition sites.

## Author Contributions

PR, JG, and AL planned the experiments. PR, JG, AA, and TR performed the experiments. PR, JG, and AL analyzed data. JG and AL wrote the manuscript.

## Conflict of Interest Statement

The authors declare that the research was conducted in the absence of any commercial or financial relationships that could be construed as a potential conflict of interest.
