# Supplementary material for: Low Affinity DnaA-ATP Recognition Sites in E. coli oriC Make Non-equivalent and Growth Rate-Dependent Contributions to the Regulated Timing of Chromosome Replication
Source: Front Microbiol. 2018 Jul 26;9:1673. doi: 10.3389/fmicb.2018.01673 (PMC6070618; doi:10.3389/fmicb.2018.01673)
Supplement: Supplementary file 1 [file Image_1.PDF]

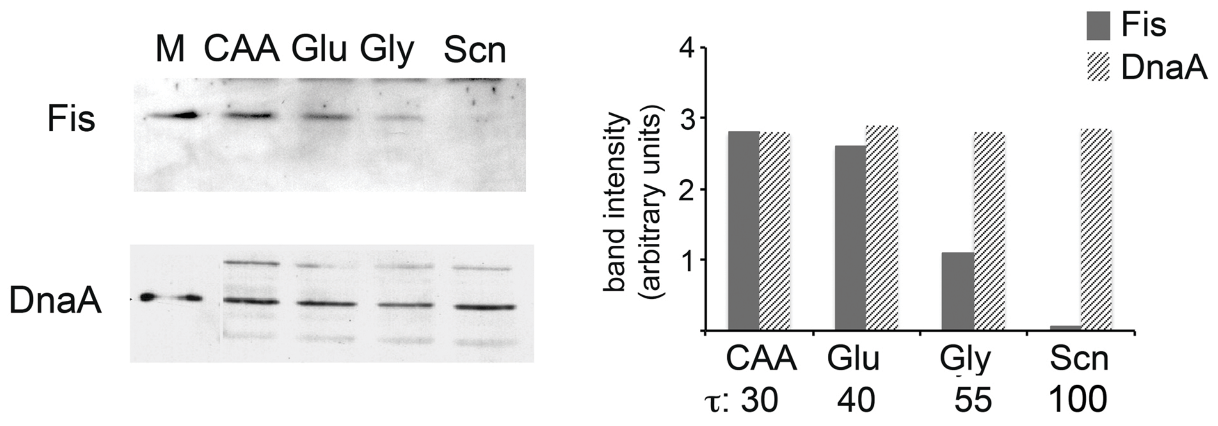

Figure S1. Cellular levels of Fis decrease as a function of growth rate. Cell lysates of *E. coli* growing exponentially in minimal media supplemented with glucose and casamino acids (CAA, generation time of 33 minutes); glucose (Glu, generation time of 40 minutes), glycerol (Gly, generation time of 60 minutes) and succinate (Scn, generation time of 100 min) were separated by electrophoresis on denaturing polyacrylamide gels and levels of Fis and DnaA were compared using immunoblots probed with anti-Fis or anti-DnaA antibodies. Purified protein was used as a size marker for the proteins. The blots were quantified using BioRad Quantity One software (right panel).

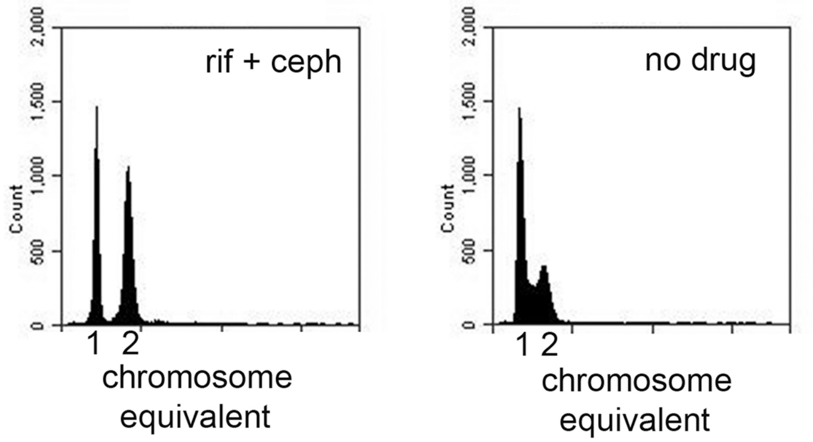

Figure S2. ***E. coli*** **cells growing with succinate as the carbon source do not exhibit rifampicin-resistant initiations.** MG1655 growing in minimal medial supplemented with succinate after treatment with cephalexin and rifampicin, or before drug treatment, were fixed and processed for flow cytometry as described in Material and Methods. The DNA histograms show the number of chromosome equivalents, corresponding to the number origins in the cells.
